# Rhabdomyosarcoma in Adults: A Retrospective Analysis of Case Records Diagnosed between 1979 and 2018 in Western Denmark

**DOI:** 10.1155/2021/9948885

**Published:** 2021-08-30

**Authors:** Vivi-Nelli Mäkinen, Akmal Safwat, Ninna Aggerholm-Pedersen

**Affiliations:** ^1^Faculty of Health, Aarhus University, Aarhus, Denmark; ^2^Department of Oncology and Danish Center for Particle Therapy, Aarhus University Hospital, Aarhus, Denmark; ^3^Department of Oncology, Aarhus University Hospital, Aarhus, Denmark

## Abstract

**Introduction:**

Adult rhabdomyosarcoma is a rare tumour that has an inferior survival compared to the paediatric patient population. The reason for this consistently worse outcome remains mostly unknown. It has been suggested that this disparity may be related to biological and/or treatment-related factors, which in the literature has been shown to be distributed differently among paediatric and adult patients. The aim of this study was to clarify treatment outcome and clinicopathological factors for adult patients with rhabdomyosarcoma that were treated in Aarhus, Denmark, since 1979.

**Methods:**

By searching the Aarhus Sarcoma Registers, data for all rhabdomyosarcoma patients, aged 18 years or more, between 1979 and 2018, were retrieved and analysed.

**Results:**

Data from 50 patients were collected. No patients were lost to follow-up. For the entire cohort, 5- and 10-year overall survival rates were 30% and 18%, respectively. The median age was 46.5 years, and the median overall survival was 2.3 years. Tumour histology was embryonal 18%, alveolar 22%, pleomorphic 44%, and not otherwise specified 16%. The tumour site was unfavourable in more than 80% of the patients. Significant factors associated with inferior overall survival were histology and disease stage, although histological subtype was not significant in the multivariate model. Five-year overall survival was 40% for localised disease versus 15% for metastatic disease.

**Conclusion:**

Rhabdomyosarcoma in adults has a poorer prognosis than paediatric rhabdomyosarcoma and other high-grade sarcomas in adults. Adult rhabdomyosarcoma should continue to be treated aggressively, but new and tailored treatment strategies are needed to improve the long-term outcome. Previous predictors of poor survival in paediatric patients were valid in adults except for age, site (favourable versus unfavourable), and tumour size.

## 1. Introduction

Rhabdomyosarcoma (RMS) is a malignant tumour of soft tissue assumed to originate from the same pluripotent mesenchyme cell as striated skeletal muscle. RMS can arise in many different sites throughout the body and is classified into three major histological subtypes: pleomorphic rhabdomyosarcoma (PRMS), embryonal rhabdomyosarcoma (ERMS), and alveolar rhabdomyosarcoma (ARMS). The pleomorphic subtype is currently defined as a high-grade sarcoma composed of undifferentiated round and spindle cells. In comparison, ovoid to spindle cells are usually described in embryonal, and round and ovoid cells are found in alveolar RMS, which is also characterised by a possible FOXO1-PAX3/7 translocation and aggressive behaviour [1–3].

Although rhabdomyosarcoma is a rare malignancy, it is the most common soft tissue sarcoma in paediatric patients, as more than 50% of the cases were diagnosed before ten years of age. In contrast, it is exceedingly rare in adults. Due to this rarity of adult-type rhabdomyosarcoma, information regarding its clinical and biologic characteristics is limited and no clinical trials specifically for adult rhabdomyosarcoma have been performed [4, 5].

The current therapeutic approaches for newly diagnosed paediatric patients are outlined in the Intergroup Rhabdomyosarcoma Study Group-V (IRS-V) study and European paediatric Soft tissue sarcoma Study Group (EpSSG), in which a multidisciplinary risk-adapted treatment is recommended [6]. As a result, the prognosis for children with rhabdomyosarcoma has improved considerably. Currently, the five-year overall survival rates have increased to around 70% for children with the nonmetastatic disease [2, 7].

Unfortunately, these results have not been translated into better long-term survival rates for adult patients as data from retrospective studies consistently demonstrated inferior survival [4, 8]. Five-year overall survival rates from single-institution studies were in the range of 21% to 53% (Table 1).

The reasons for this consistently worse outcome remain mostly unknown. One explanation for the lower survival rates seen in adults is an increased incidence of adverse prognostic factors such as unfavourable primary sites and higher rates of regional and distant spread [2]. Because of these factors, adults are already from the time of diagnosis assigned to high-risk and very high-risk groups with inferior prognoses according to EpSSG [6]. The histology of adult rhabdomyosarcoma has also been described as unfavourable with a predominance of pleomorphic histology, which is a rare disease, mainly of adults, and associated with a potential aggressive clinical course. This makes it difficult to compare data on paediatric and adult RMS, as there is a disproportional incidence of PRMS in adults [17]. The histological characteristics of pleomorphic RMS may offer some explanations for its aggressive behaviour and its poor response to conventional chemotherapy [18]. Recent studies have also focused on alveolar molecular characterisation based on a recurrent chromosomal translocation that fuses two transcription factor-encoding genes (PAX3/7 and the FOXO1), which have been implicated in metastatic presentation, recurrence, and resistance to therapy [19, 20]. Notably, the fusion-positive cases have shown a relationship with higher age at diagnosis and have a low prognostic significance [6, 21].

To date, no standardised protocol specifically for adult patients has been established mainly because of the limited number of studies for this group, and adults are therefore currently treated according to paediatric regimens [5, 6]. Issues in tolerating this intensive regimen might have a role in the different outcomes according to age, and it has been suggested that suboptimal treatment in adults as compared to children accounts for the low overall survival seen in adult rhabdomyosarcoma [3, 5, 13]. Therefore, the poorer survival of adult rhabdomyosarcoma is likely to be multifactorial [4, 16, 18].

This cohort study aims to retrospectively analyse case records from the population-based Aarhus Sarcoma Registries to determine the clinicopathological features and outcomes for patients with adult rhabdomyosarcoma in Western Denmark.

## 2. Materials and Methods

### 2.1. Data Collection

A retrospective cohort study was conducted by searching the Aarhus Sarcoma Registers in spring 2020 in order to identify patients diagnosed with rhabdomyosarcoma between 1979 and December 2018 (*n* = 92). These databases consist of both clinical and survival data of all sarcoma patients from Western Denmark.

Inclusion criteria were ≥ 18 years of age and an RMS diagnosis. Therefore, all patients under 18 were excluded from the study (*n* = 35), as 17 years of age is the cut-point used at our institution for paediatric patients. We excluded seven patients with disease that was wrongly labelled as RMS.

This left 50 patients eligible for the study. We collected data on baseline characteristics including age, gender, primary tumour location, tumour size, disease stage, metastatic involvement sites, and outcome. The disease stage was determined by X-ray, CT, MRI, or PET-CT. The absence of distant metastases defined the localised disease. All cases labelled as metastatic disease were staged after CT imaging. As each citizen in Denmark is given a unique 10-digit personal registration number, complete follow-up is enabled, unless the patient had moved abroad.

### 2.2. Pathology

Aarhus University Hospital is one of the two tertiary sarcoma centers located in Denmark and a European reference center for sarcomas. Since the establishment of the center in 1979, only pathologists with the necessary expertise on classifying sarcomas have been working at the center. We believe that patients have received the most accurate histological classification at the time of their diagnosis and the pathology in the dataset was not rereviewed.

### 2.3. Statistical Analysis

The primary outcome used in this study was overall survival. Overall survival was defined as the time between diagnosis and death or censoring at the date of the last visit. Overall survival curves were constructed using the Kaplan–Meier method to assess the probability of survival without event during the follow-up period. Patients still alive at the end of the study or lost to follow-up were censored at the last follow-up date. Patient outcome was assessed according to the following clinicopathological variables: gender, age, disease stage, tumour size, histologic subtype, and primary site. Tumour size was divided into two groups with tumours < 5 cm and ≥ 5 cm, respectively, as the most recent EpSSG has demonstrated < 5 cm as favourable [6]. Age was treated as a continuous variable. A univariate analysis was conducted using Cox's proportional hazards regression method. A multivariate analysis of prognostic factors was only possible for a limited number of factors because of the sample size. A *p* value < 0.05 was considered significant. All *p* values were two-sided. All statistical analyses were performed with Stata software version 15.1 (Stata Corporation, College Station, Texas, USA).

## 3. Results

### 3.1. Patient Demographics

The cohort included 50 patients, 18 years of age and older, with a reported diagnosis of rhabdomyosarcoma. The median age was 46.5 years with a range of 18–78, while 55% of patients were 40 years or older. The mean age was 45.4 years (standard deviation, 19.4). Females and males were represented equally. Of the 50 patients, 20 patients (40%) had metastatic disease at the time of diagnosis. The average follow-up time was 48 months.

### 3.2. Tumour Characteristics

The primary tumour site was head and neck in five cases (10%), intrathoracic, abdominal, and retroperitoneum organs in 14 (28%), extremity in 24 (48%), and genitourinary in seven (14%). On classifying tumours by favourable or unfavourable prognostic sites (according to the most recent EpSSG publication) [2], nine patients had favourable sites, and 40 patients had unfavourable sites. Of the 33 patients with tumours > 5 cm, a third was localised in the extremities, classified as an unfavourable site. The average tumour size was 7.5 cm (range, 1–30 cm).

Pleomorphic rhabdomyosarcoma was the most common histologic subtype, accounting for 22 patients (median age, 54 years; range 18–77). Compared to the subset of 28 patients with alveolar, embryonal, or not specified histotype, there was no difference in sex proportion.

The pleomorphic subtype was dominant in the extremities (43%) and one-third of the visceral tumours were pleomorphic subtype. Patient and tumour characteristics for both disease stages are summarised in Table 2. No information regarding translocations was available in the registers.

### 3.3. Treatment

The majority of the patients (*n* = 35, 70%) were treated with a treatment strategy consisting of either one modality or a combination of two of the following modalities: surgery, radiation, and chemotherapy, thus leaving a subgroup of 11 patients who were treated with a multimodality approach (consisting of all three modalities) and four patients who were not treated. These four patients presented with an unfavourable histologic subtype, and three-quarters had metastatic disease with pulmonary involvement.

Surgery was the primary treatment (*n* = 8) among the patients, who were only treated with one modality.

The majority of the patients in the cohort (*n* = 38, 76%) underwent surgical resection. Of the subset of twelve patients who did not undergo surgery, four were not treated; four patients were treated with chemotherapy and radiation and the remaining four with either chemotherapy or radiation.

Chemotherapy was administrated to 27 patients (54%). The most commonly used chemotherapeutical agents were vincristine, actinomycin *D*, ifosfamide, and doxorubicin. The median age for patients receiving chemotherapy was 32 years (range, 18–75) and 56 years (range, 24–78) for those who did not. Only six patients with PRMS were treated with systemic chemotherapy when subgrouping the cohort.

External beam radiation therapy was included in the treatment of 24 patients. Median doses were 50 Gy and 35.5 Gy for patients receiving treatment with curative or palliative intention, respectively.

### 3.4. Management of Localised and Metastatic Disease

#### 3.4.1. Localised Disease

56% of the patients (*n* = 18) with localised disease were treated with a combination of more than two of the following modalities: surgery, chemotherapy, and radiotherapy. Surgery was a part of the treatment in over 90% (*n* = 27) of patients with localised, nonmetastatic disease.

Six patients were treated with all three modalities. Patients receiving multimodality treatment were on average younger than those who did not (mean age, 40 versus 51 years).

Of the patients with localised disease who were treated with one modality (*n* = 11), nine underwent surgery (73%). In contrast, chemotherapy administration was limited for patients with localised disease, as nineteen patients (63%) did not receive it as a part of the treatment strategy.

#### 3.4.2. Metastatic Disease

The majority of the patients with metastatic disease (*n* = 14, 66%) were treated with more than one modality and five received treatment combination of all three modalities. Sixteen patients (80%) received chemotherapy; nine (43%) were treated with radiation; and 11 (52%) underwent surgery. A combination of chemotherapy and surgery or chemo- and radiation therapy was almost equally utilised (five versus three patients).

Three patients were only treated with one modality, either chemotherapy or surgical resection of the primary tumour. Data regarding tumour response to chemotherapy were only available in four cases.

### 3.5. Survival Analysis and Outcome

21 (41%) of the 50 patients relapsed during the study period: six versus 15 patients with nonmetastatic disease. Eight patients had a local relapse at the primary tumour site; two had a local relapse the regional site; and eleven (52%) patients had metastatic relapses. The median time to first relapse was 14 months (range 3–168 months). The median follow-up was 26 months (range 0–343) for all patients and 152 months (range 57–343) for survivors. Three patients had a second relapse. At the time of analysis, 40 patients had died from their disease. No patients were lost to follow-up.

**Figure 1:**
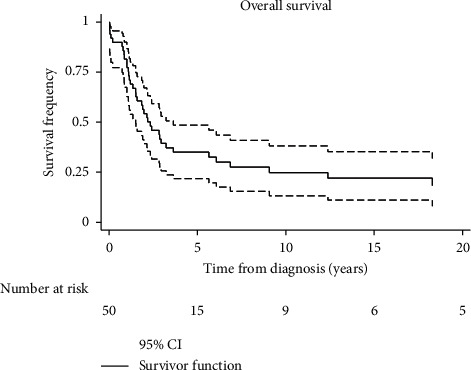
Kaplan–Meier overall survival rates for all patients. The dotted line is the 95% confidence interval.

Five- and 10-year overall survival for the entire cohort were 30% and 18%, respectively (Figure 1). There were no outcome differences between patients treated before and after the year 2000.

**Figure 2:**
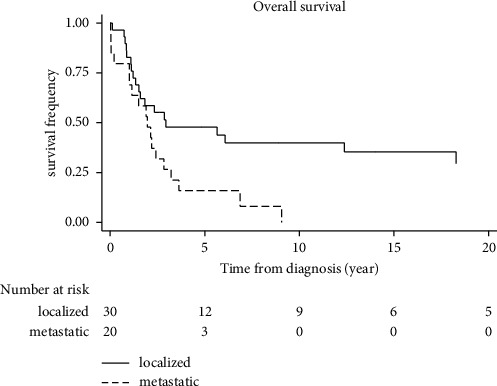
Kaplan–Meier overall survival rates stratified by disease status (localised versus metastatic).

The median overall survival was 2.3 years (95% CI: 1.5–3.6). Five-year OS was 15% in the subgroup of 20 patients with metastatic disease and increased to 40% for patients within the subgroup who presented with localised disease at the time of diagnosis (Figure 2). For patients receiving chemotherapy, the mean follow-up time was 40 months and 65 for those who did not.

We subgrouped the cohort according to the different treatment modalities. Our data suggest that patients receiving surgery and radiation therapy performed better than patients treated with surgery alone or multimodal therapy. Similarly, patients with wider surgical margins had better survival estimates. However, it is important to notice that there might be a difference in, for example, histological subtypes, disease stage, performance status, anatomical location, and age when selecting a patient for multimodal therapy versus surgery or radiation therapy.

Prognostic factors of improved disease-specific survival by univariate analyses (Table 3) were histology (*p*=0.042) and disease extent (*p*=0.016).

When the cohort was divided by the anatomic site (favourable versus unfavourable by following recent EpSSG), there was no statistically significant difference (*p*=0.18). At the time of the initial presentation, small tumours (< 5 cm) were not associated with more prolonged survival (*p*=0.39).

Age was treated as a continuous variable.  Table 4 presents the results. Data suggests that, for every 1-year increase in age, the risk of death is increased by a factor of 1.015. However, the *p* value failed to reach a significant level.

#### 3.5.1. Multivariate Analysis

Table 4 presents the results of the multivariate logistic regression analysis for survival, but age and histological subtype (ARMS and ERMS versus NOS and PRMS) did not reach a significant level in this multivariate model.

## 4. Discussion

This retrospective cohort study included 50 adults in the age range of 18–78 years diagnosed with rhabdomyosarcoma, who were registered in the population-based Aarhus Sarcoma Registries from 1979 to 2018. Among the 50 patients, 5-year overall survival was in the range of 30%. This rate is lower than rates reported in paediatric trials but closely parallels with results from previously published studies in Table 1. The studies in the table also included adults with PRMS, associated with a very poor prognosis. Our analysis confirmed that one reason for the poor prognosis in adult rhabdomyosarcoma patients is an unfavourable clinical presentation, such as distant metastases at the time of diagnosis, unfavourable histology, and a highly heterogeneous treatment approach. We did not find any difference in overall survival between patients treated before and after 2000, which might be due to patient demographics, tumour characteristics, and lack of evolution in treatment strategies. This could also be the explanation for why patients receiving surgery and radiation therapy performed better than patients treated with surgery alone or multimodal therapy. Numerous demographic characteristics were different from previous adult studies, that is, an older patient population, a larger number of relapses, and an equal male-to-female ratio. In our analysis, patients with adult rhabdomyosarcoma did not show a predilection for males, as noted in the paediatric literature, but the effect of sex on survival remains unclear in the paediatric literature [11]. The majority of the studies listed in Table 1 reported a median age of < 30, whereas 46 years of age was the median in our cohort. Age itself has previously been associated with poor disease outcomes, as IRS-V study reported a significantly improved 3-year failure-free survival rate among children less than ten years of age [7]. Still, there are studies on adult rhabdomyosarcoma reporting conflicting results regarding age being a predictor of survival [2]. Little et al. [8] and Esnaola et al. [11] noted that increasing age was not associated with inferior survival, as opposed to recent studies by Dumont et al. [10] and Gerber et al. [2] who reported age as significant for overall survival in nonmetastatic adult patients. The reasons for this age effect are still unclear but it could be caused by differences in the distribution of histological subtypes or lower adult patient tolerance for intensive chemotherapy [8]. In our study, age was not a significant predictor of overall survival for the entire cohort. Tumour size has previously been associated with poor survival [7]. In our study, increasing tumour size was not significantly associated with worse survival on univariate analysis. Sample size limitation could cause the fact that tumour size failed to reach statistical significance. Consistent with current literature stating that PRMS is mainly a subtype seen in adults-RMS patients, it was also the most common histological subtype in our study. This appears to be an adverse prognostic factor in both paediatric and adult rhabdomyosarcoma. In our experience, histologic subtype did also appear to significantly impact survival (*p*=0.042), when subgrouped into favourable and unfavourable subtypes, according to the recent EpSSG. Moreover, histologic subtype did vary according to anatomic site and age in agreement with previously published reviews. Further investigation of histologic subtype's prognostic importance is needed due to the small sample size in this study. Because of the small number of patients with ARMS, we did not analyse whether younger patients with ERMS versus ARMS had a statistically better outcome, but we believe that younger patients with favourable histological subtypes theoretically would most likely have better outcomes. Previous studies in the literature have noted that adult rhabdomyosarcoma arises predominantly in the extremities [1, 4]. These findings are aligning with our results, as the primary tumour was mainly located in the extremities, accounting for 44% of our cases. In general, adult rhabdomyosarcoma in the extremities has been associated with a poor prognosis and, therefore, counted as an unfavourable site [1]. In our analysis, anatomical location divided into “favourable” and “unfavourable” failed to reach statistical significance; this was also the case in a study by Hawkins et al. [1]. Nevertheless, the primary tumour location is still clinically meaningful, as tumours occurring in, for example, the retroperitoneum, can become quite large before producing signs and symptoms [22]. The reason for the low percentage of patients receiving chemotherapy in our study (54%) (Table 5) could be the predominance of pleomorphic subtype in our cohort (44%), as the majority of this subgroup would be treated as nonrhabdomyosarcoma with surgery only or with a combination of radiotherapy, as pleomorphic rhabdomyosarcoma has been shown not to be as responsive to chemotherapy as the embryonal or alveolar subtypes [18, 22]. Secondly, patients who had chemotherapy as a part of their treatment were on average almost 25 years younger than those who did not. Therefore, these older adults could have been more likely to receive subtherapeutic dosing of chemotherapy than younger patients due to toxicities and complications. There could be comorbid diseases and loss of organ function explaining the pattern of use [23]. The differences in adjuvant therapy could also point towards a lack of uniformity of treatment over these years, but it could also be a well-considered decision not to include chemotherapy in the regime. Balancing the quality of life versus potential side effects and short life expectancy may have been considered. This reflects an urgent need for new therapeutic options for adult RMS patients in order to optimize the balance between cost of cure and potential benefit of RMS treatment [21]. On a similar note, various authors have reported that adults with rhabdomyosarcoma tended to be treated with various chemotherapy regimens (ranging from multiagent paediatric chemotherapy schedules to single-agent doxorubicin), reflecting a lack of consensus on a standardised approach to adult rhabdomyosarcoma [2, 3]. This was also the case in our study, making it difficult to compare various groups concerning chemotherapy and treatment modalities in general and hard to analyse data with statistical methods. The variation may be explained by the long study period reflecting different treatment protocols in time and the lack of consensus. Unfortunately, the current dataset does not provide detail on chemotherapy regimens, and thus whether these guidelines have affected OS rates is unknown. Nonetheless, in a recent study by Bergamaschi et al. [16], the authors emphasised the idea that adult patients treated in line with paediatric strategy could potentially improve adult rhabdomyosarcoma outcomes. Studies by Ferrari et al. [12] and Khosla et al. [15] demonstrated a better survival and local control in adults treated with multimodality therapy on lines of paediatric rhabdomyosarcoma but also raised the question of whether adults might tolerate treatments designed for children to a lesser degree [12]. Therefore, it has been questioned whether rhabdomyosarcoma patients could benefit from molecularly targeted and immunotherapeutic approaches, potentially reducing the treatment-associated toxicities caused by current chemotherapy and radiation therapy [19, 21]. The heterogeneity in the management of rhabdomyosarcoma may have affected the outcome of the studied patients. Recently, there has been growing attention towards understanding the molecular biology of rhabdomyosarcoma to help to refine risk stratification and appropriately tailor treatment for these patients. In particular, identifying the PAX/FOXO-1 fusion with molecular testing is now recommended on all patients with alveolar or embryonal histology, as results may impact treatment decisions and because fusion status replaces histology as a stratifying factor [18]. In our case, information regarding gene fusion status was not available. Still, it is not unreasonable to believe that some of the patients with a reported alveolar-subtype conceivably were fusion-positive, thus explaining the outcome for these patients, as the fusion gene products possess transcriptional activity and are involved in the tumourigenesis of alveolar rhabdomyosarcoma [6].

## 5. Bias

As previously noted, we did not rereview the histological diagnoses. Therefore, there could be a potential misclassification of the tumour histology, resulting in an inaccurate diagnosis. Should there be any misclassification, it is believed to be nondifferential, as the probability of individuals being misclassified is equal across all groups in the study. Still, the potential impact of this would be the risk of including patient records, where the histological classification is not RMS. This could introduce bias and a deviation of the observed overall survival rates and consequently threaten the internal validity of the estimates.

Selection bias is considered to be a minor problem. Firstly, the data source is a population-based database, meaning that all sarcoma patients in the uptake area will be referred regardless of the severity of their disease. Secondly, nonresponse selection bias is thought to be almost nonexistent as patients are registered as part of the treatment protocol and not asked to be a part of the registry (with a chance of deckling), preventing the risk of bias.

## 6. Limitations

Our study is firstly limited by its retrospective nature and a small number of patients treated over a long period. Notable deficiencies were the lack of data on information regarding the fusion gene status, details on gene translocation, dose of radiation, and chemotherapy treatment cycles and effects. Because of the small sample size, further division of the cohort into smaller groups often yielded groups with less than 10 events. Still, we performed multivariate analyses, but due to the sample size, one should be cautious when interpreting the results. The relatively small number of patients in our study limited the potential to identify prognostic variables and resulted in numerous subgroups of ≤ 2 patients. We could not report additional data regarding these patients due to Danish law regulations and requirements. There are limitations regarding the reliability of all register-based studies, but Aarhus Sarcoma Registers are reported to be a valuable population-based tool for epidemiological research [24].

## 7. Conclusions

In the current study, we confirmed that the outcome for adults with rhabdomyosarcoma appears to be inferior compared to children, which is in line with previously published studies. The reasons are likely multifactorial and may include overrepresentation of pleomorphic rhabdomyosarcoma, limited access to clinical trials, and poor adherence to aggressive treatment regimens. Additional studies on treatment approaches, variables, and adult rhabdomyosarcoma's biology are still needed to further clarify differences between adult and paediatric rhabdomyosarcoma.

## Figures and Tables

**Table 1:** Reported 5-year survival rates for adult rhabdomyosarcoma.

Author (ref.)	No. of patients	Time period	Median age (years)	5-year survival rate
Lloyd et al. [9]	54	1950–1978	—	21%
Dumont et al. [10]	239	1957–2003	19	44%^*∗*^
Little et al. [8]	157	1960–1988	—	44%
Esnaola et al. [11]	39	1973–1996	27	31%
Sultan et al. [4]	1071	1973–2005	> 19	27%
Ferrari et al. [12]	171	1975–2001	27	40%
Hawkins et al. [1]	84	1982–1999	31	35%
Bompas et al. [13]	449	1981–2014	26	30%
Stock et al. [14]	57	1985–2008	59	52,6%
Gerber et al. [2]	148	1990–2011	28	34%
Khosla et al. [15]	25	2000–2009	19	45%
Bergamaschi et al. [16]	95	2002–2015	27	40,3%
Drabbe et al. [17]	66	1990–2020	28	27%

**Table 2:** Baseline clinical characteristics at diagnosis of localised or metastatic disease.

	Localised disease (*N* = 30)	Metastatic disease (*N* = 20)
Median age (SD)	43 (18.5)	49 (21.1)

Sex		
Female	14	11
Male	16	9

Median tumour size, cm (range)	6 (1–27)	10 (4–30)

Anatomical location of the primary tumour		
Head and neck (nonparameningeal and parameningeal)	4	≤ 2^*∗*^
Intrathoracic, abdominal, and retroperitoneal organs	7	7
Extremities	14	10
Genitourinary	5	NA^*∗*^

Site^*∗∗*^		
Unfavourable	22	18
Favourable	7	NA^*∗*^

Metastatic site		
Lung	0	9
Liver	0	≤ 2^*∗*^
Bone	0	≤ 2^*∗*^
Others	0	8

Treatment intent		
Curative	13	9
Palliative	5	6

Site: favourable sites are GU including bladder prostate, head and neck nonparameningeal, orbit, and biliary primaries; unfavourable sites are all other sites including NOS and extremities [8]. Histologic subtype: favourable: embryonal; unfavourable: alveolar, pleomorphic, and not otherwise specified [2]. ^*∗*^According to the Danish law, it is not legal to report on individual patients without the patients' signed consent. Therefore, if a cell includes ≤ 2 patients, the cells with the lowest number of patients have been blinded.

**Table 3:** Univariate analysis of factors potentially affecting overall survival.

Variable	Hazard ratio	95% CI	*P* value
Gender			
Female	1	—	—
Male	0.78	0.4–1.5	0.46

Age	1.015	0.99–1.031	0.076

Histology			
Favourable	1	—	—
Unfavourable	2.95	1.04–8.36	0.042^*∗*^

Tumour size			
< 5 cm	1	—	—
≥ 5 cm	1.44	0.63–3.33	0.39

Disease stage			
Localised	1	—	—
Metastatic	2.3	1.2–4.5	0.016^*∗*^

^*∗*^Statistically significant site: favourable sites are GU and head and neck nonparameningeal; unfavourable sites are all other sites, not otherwise specified, and extremities. Histologic subtype: favourable: embryonal; unfavourable: alveolar, pleomorphic, and not otherwise specified [2].

**Table 4:** Multivariate analysis.

Variable	Hazard ratio	95% CI	*P* value
Age^*∗*^	1.014	0.99–1.03	0.096
Histological subtype^*∗∗*^	2.73	0.96–7.73	0.058

^*∗*^Age as a continuous variable. ^*∗∗*^Histology (ARMS and ERMS versus NOS and PRMS).

**Table 5:** Histologic subtype and the administration of chemotherapy.

Chemotherapy	Histological subtype				Total
	NOS	ARMS	ERMS	PRMS	
Yes	5	9	7	6	27
No	3	2	2	16	23
Total	8	11	9	22	50

## Data Availability

The data are not available due to ethical restrictions. Due to the nature of this research, participants of this study did not agree for their data to be shared publicly and it could compromise research participant's privacy and consent.
